# Medium-term Outcomes of Cemented Prostheses and Cementless Modular Prostheses in Revision Total Hip Arthroplasty

**DOI:** 10.1038/srep02796

**Published:** 2013-09-30

**Authors:** Long Wang, Pengfei Lei, Jie Xie, Kanghua Li, Zixun Dai, Yihe Hu

**Affiliations:** 1 Department of Orthopedics, Xiang Ya Hospital Central South University, Changsha, Hunan, China

## Abstract

There is an increasing trend towards cementless modular femoral prostheses for revision hip replacement surgery, especially in patients with severe proximal femoral bone defects. However, for minor femoral bone defects, the benefit of cementless modular is not clear. We designed a retrospective cross-sectional study to compare outcomes of the two femoral implant designs. There were no significant differences in terms of visual analog pain scores, Harris hip scores, femoral bone restoration, stem subsidence, leg length correction, or overall complication rate. Three femoral reoperations (11%) occurred in the cemented group, and two (9%) in the cementless modular group. One femoral stem re-revised (4%) in the cemented group due to recurrent deep infection. Five-year survival for femoral reoperation was 88.2% for patients with the cemented implant and 91.3% for cementless group. Both groups had good clinical and radiological outcomes for femoral revision in patients with minor femoral bone defects during medium-term follow-up.

Revision total hip arthroplasty (THA) is technically challenging due to varying amounts of bone loss, altered femoral geometry, differing degrees of fixation of the implant, and poor bone stock. Given the unpredictable nature of revision THA and usually suboptimal results compared to primary surgery, numerous implant designs, cementless and cemented fixations, and surgical techniques have been investigated and performed with intent to improve outcomes of the procedure.

Femoral stem revisions were initially performed using long cemented stems, the procedure technically similar to conventional primary cemented THA. However, it was observed that a stable fixation can be difficult to achieve with cement especially in those patients who have poor femoral bone stock^1^. Some studies showed good clinical results during femoral revision when a cemented stem was used in conjugation with an impacted bone graft^2^,^3^,^4^. However, this technique was not widely used clinically because of a high intraoperative and postoperative femoral fracture rate, frequent postoperative subsidence, and prolonged operative time requirements^4^,^5^,^6^,^7^,^8^.

During the last decade the cementless stem, especially the cementless modular stem, has been increasingly used in femoral revision. The modular femoral component works on the principle of distal fixation in the diaphysis of the femur. This facilitates the intraoperative adjustment of leg length, femoral offset, and neck anteversion to maximize implant stability and hip kinematics.

The Link MP stem used in this study is an uncemented stem whose design is based on the principles of distal fixation. Its modularity enables the surgeons to overcome unpredictable bone deformities intraoperatively, and bypasses the deficient proximal femur to achieve fixation distally. Excellent clinical results in several previous studies^9^,^10^,^11^ encouraged the application of this modular femoral stem in hip revision.

The objective of this study was to evaluate whether the cementless femoral modular stem (Link MP, Waldemar Link, Hamburg, Germany) is better than the cemented stem (Lubinus SP II stem, Waldemar Link, Hamburg, Germany) for THA revision in patients with minor bone defects, evaluated at short- to medium-term follow-up. Our evaluations of the stem included three important outcome goals for revision hip arthroplasty: (1) improving hip function and relieving pain, (2) avoiding re-revisions and complications, and (3) the preservation and restoration of bone stock.

## Results

### Clinical results

At the last follow-up, the mean postoperative HHS was 79.2 (range, 32–100) in the cemented stem group and 83.9 (range, 61–100) in the cementless modular stem group. The postoperative VAPS scores averaged 0.7 (range, 0–4) in the cemented group and 0.5 (range, 0–3) in the cementless group. The postoperative HHS and VAPS both showed significant improvement in clinical results from the preoperative scores (*P* < 0.01). Although the improvement of the postoperative HHS and VAPS favored the cementless modular stem group, the differences (HHS, *P* = 0.480; VAPS, *P* = 0.135) were not significant (Table 2).

Mild thigh pain occurred in 1 (4%) patients in the cemented group and 2 (9%) patient in the cementless group during movement. However, none of these required nonsteroidal anti-inflammatory drugs or opioid analgesics for pain relief. Delayed wound healing was observed in only one patient (4%) in the cementless group.

### Radiographic follow-up

The postoperative radiographs evaluating the biologic fixation^12^ of the cementless modular stems revealed that 21 (91%) had osseointegration, and 2 (9%) showed stable fibrous fixation. In the cemented group, 24 (85%) were classified as well-fixed, 3 (11%) were possibly loose and one (4%) was definitely loose^13^. The only definitely loose prosthesis was re-revised because of recurrent deep infection.

Compared to the immediate postoperative radiographs, the median stem subsidence was 2.0 mm (range, 0–8 mm) in the cemented group and 1.4 mm (range, 0–9 mm) in the cementless group at last follow-up. Improvement of mean stem subsidence favored the cementless modular stem group, but the difference (*P* = 0.304) was not statistically significant. There was one (4%) stem in the cemented group and 2 (9%) stems in the cementless group that had subsidence > 5 mm. Only one cemented stem subsided progressively and was re-revised in the first year after surgery because of recurrent deep infection. The other two patients with subsidence > 5 mm were asymptomatic and showed no further progression of the subsidence at the last follow-up.

The median LLD preoperatively was 3.8 mm (range, 0–19 mm) in the cemented group and 5.7 mm (range, 0–26 mm) in the cementless group. Postoperatively, the LLD was 2.4 mm (range, 0–14 mm) in the cemented group and 2.8 mm (range, 0–13 mm) in the cementless group. Preoperatively there were 6 (21%) patients in the cemented group who had LLD > 5 mm, in contrast to 9 (39%) patients in the cementless group. Postoperatively there were 5 patients with LLD > 5 mm in each group at the last follow-up. The results showed statistically significant improvement in LLD in both groups, especially in the cementless group (cemented, *P* = 0.001; cementless, *P* = 0.004).

Comparison of the immediate postoperative and last follow-up radiographs revealed that 7 hips (25%) had femoral bone restoration. Fifteen hips (54%) had constant defects and 6 hips (21%) showed increasing defects in the cemented stem group, while 11 hips (48%) had femoral bone restoration, 8 hips (35%) had constant defects, and 4 hips (17%) had increasing defects in the cementless modular stem group. The difference was not statistically significant between the two groups (*P* = 0.228).The increasing bone defects were mostly seen around the lesser trochanter. Four patients (14%) in the cemented group and 2 (9%) patients in the cementless modular group had notable radiolucencies (*P* = 0.678), but all of these were non-progressive with a clinically stable stem.

### Complications and survival rate

Intraoperative femoral fracture occurred in 3 (13%) hips in the cementless modular stem group; one during the old stem or cement removal, two during stem implantation. All the fractures were treated with cerclage wires or cables, and in one hip a stem longer than preoperatively planed was used to obtain better distal fixation. All the fractures healed without any further complications and the postoperative radiographs showed stable fixation of the stem.

**Figure 1:**
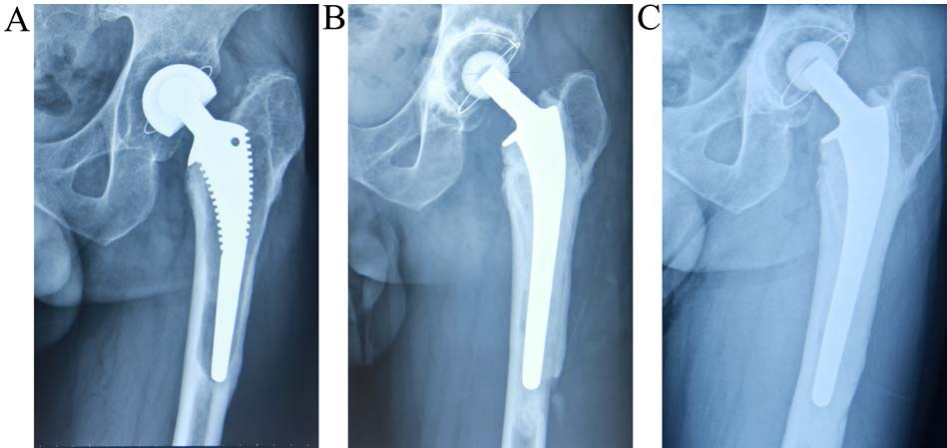
Serial radiographs of a 69-year-old man who underwent revision with the cemented stem (Lubinus SP II). (a) Radiograph before revision, showing a loosened stem. (b) Radiograph immediately after revision with the Lubinus SP II cemented femoral stem. Cortical perforation occurred during the stem implantation. (c) Radiograph 7 years after revision with the Lubinus SP II stem. Cortical perforation healed without any progression of fracture.

Only one hip (4%) in the cementless group dislocated during the immediate postoperative period and had recurrent dislocation in subsequent follow-ups. The case subsequently required a revision procedure and a longer head-neck was used to increase stability. Cortical perforation occurred in 2 hips (7%) in the cemented group and 3 hips (13%) in the cementless group. A longer stem than originally planned preoperatively was used in one hip in the cementless group, while the others healed without any intervention (Fig. 1). Sciatic nerve palsy appeared in one hip (4%) in the cemented group but resolved spontaneously without intervention. Deep vein thrombosis occurred in one patient (4%) in the cemented stem group and was treated with outpatient anticoagulation.

There was one femoral stem re-revision (4%) in the cemented group due to recurrent deep infection. Other reoperations included 2 acetabular revisions in the cemented stem group, and one recurrent dislocation and one periprosthetic fracture in the cementless modular stem group. The difference in reoperation rates between the two groups was not statistically significant (*P* = 0.588).

**Figure 2:**
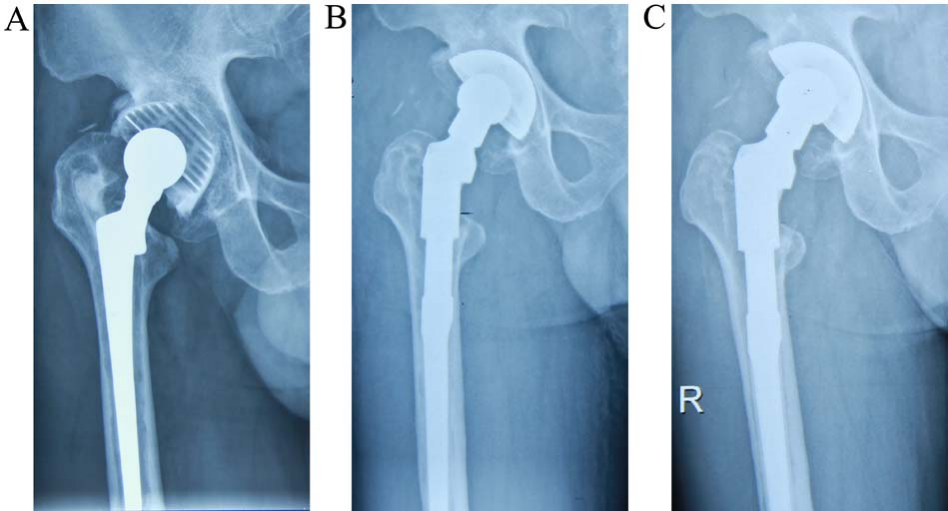
Serial radiographs of a 60-year-old man who underwent revision with the cementless modular femoral stem (LinkMP). (a) Radiograph before revision, showing a loosened stem. (b) Radiograph immediately after revision with the LinkMP modular femoral prosthesis. (c) Radiograph 4 years after the revision. The patient had a good clinical result, and the stem remains stable with no subsidence.

**Figure 3:**
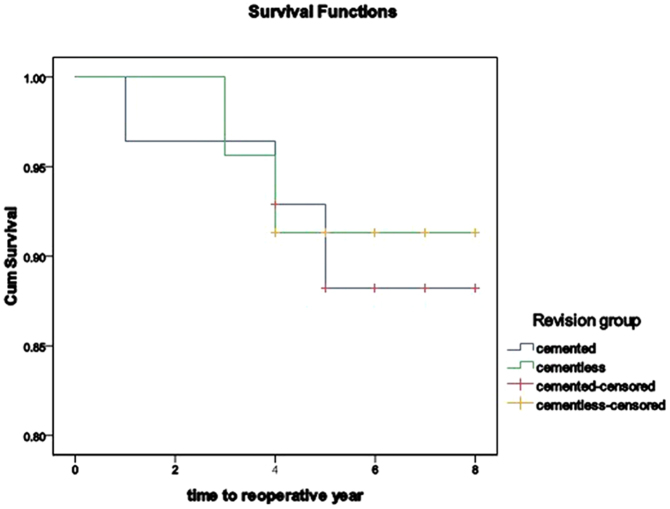
Survivorship of implants with reoperation as the end point.

Kaplan-Meier survivorship analysis was performed with 2 end points for each cohort: femoral stem re-revision and any other reoperation. The cumulative 5-year survival rate with failure defined as removal of the stem was 96.4% (95% CI, 89.5–100%) in the cemented stem group. No stem was revised in the cementless modular stem group (Fig. 2) and therefore the figure of survivorship with femoral revision could not be drawn. With any reoperation as the end point, the cumulative 5-year survival ratein the cemented group was 88.2% (95% CI, 75.5–100%) and 91.3% (95% CI, 79.7–100%) in the cementless group (Fig. 3).

## Discussion

There are varying opinions about whether uncemented implants are better than cemented for revision hip replacement. The initial reports of success for uncemented prostheses and the relatively higher failure rate of cemented implants have led many surgeons to preferentially use uncemented prostheses. The rationale for using these in case of revision is the belief that bone deficiency can be treated more appropriately with bone grafting and boney ingrowth, which is more natural biologically than bulk filling with additional cement. However, the argument against uncemented implants is that the potential for bone ingrowth from deficient areas of bone is limited, especially when the bone grafts have been interposed between the host bone and the porous surface.

The results of the present study showed that both the cemented femoral stem and the cementless modular femoral stem can be used successfully for revision THA in patients with minor bone defects. In a short- to medium-term follow-up, patients' outcomes in both groups were satisfactory in terms of improving hip function, relieving pain, and avoiding femoral re-revisions, reoperations, and complications.

The re-revision rate of 4% with cemented stems in this study was lower than the 4.6%–26% rates reported by other studies^1^,^14^,^15^,^16^. The lower re-operation rates witnessed in this series may be because only patients with minor bone defects (Paprosky types I and II) formed the study group, and only short-to medium-term follow-up results were obtained. Good clinical results and bone restitution have been reported using impacted allografts and a cemented stem for femoral revision^3^,^17^. However, patients with impaction bone-grafting in the cemented stem group were excluded from this study because the cementless modular stem was implanted without the use of this technique. In addition, frequent major subsidence^4^,^7^,^18^, and high intraoperative and postoperative femoral fracture rates^6^,^8^,^19^ have been reported widely with impaction bone-grafting in revision THA.

The cementless modular femoral component has several advantages, including distal fixation, intraoperative flexibility, and a lower modulus of elasticity, all of which have contributed to favorable results seen in studies of the last decades. Several studies showed low failure rates and early success for the Link MP cementless modular stem; the re-revision rate, ranging from 2% to 4%, was significantly lower than that for cemented implants^9^,^11^,^20^. No femoral stem re-revision (0/23) was required in our study during a mean follow-up of 5.5 years, which was in good agreement with previous findings.

In the present study, the cementless modular stem had better outcomes in terms of mean HHS, VAPS, stem subsidence, bone restoration, and notable radiolucencies than the long cemented stem. However, rate of thigh pain, restoration of the mean LLD and the incidence of overall complications favored the cemented group. Yet at combined last follow-up, the differences in these measures of outcome between the two groups were not statistically significant.

Stem subsidence has been regarded as one of the biggest risk factors for hip re-revision in several studies^21^,^22^. In the cemented femoral revision, subsidence of the cemented stem might occur when the cement mantle is thin and deficient without a rigid cortical support, and rests on a flexible support from a graft bed. An incomplete cement mantle might explain why major subsidence happens in the cemented stem^23^. Patients in this study had minor bone deficiencies with no bone graft. Thus, there was only one (4%) stem in the cemented group that had major subsidence > 5 mm, and this was due to recurrent infection. Selection of an inappropriate stem diameter and under-sizing has been found to be the main reasons for progressive subsidence with the cementless modular stem^22^. Two stems (9%) with subsidence > 5 mm in the cementless group of our study were non-progressive and subsided within the first year of the revision surgery. Our findings provide evidence that most subsidence of cementless stems occurs during the first year^24^,^25^. Since the incidence of subsidence was calculated on the basis of plain radiographic evaluation and not radiostereometric analysis, there is a chance that this may have been underreported.

Correction of LLD is important for overall patient satisfaction and hip function improvement, and is considered an important objective in revision surgery. One of the advantages of the modular stem is the flexibility to adjust leg length and restore LLD intraoperatively. LLD was greatly improved in both groups of this study after surgery, with no statistical difference between the two cohorts. However, prior to surgery the cementless group had a greater mean LLD and the LLD was >5 mm in more of the patients in this group, which should be taken into account.

The thinner and more flexible modular femoral components are assumed to stimulate regrowth of the proximal femoral bone. In the present study, a greater degree of restoration of proximal femoral bone stock was seen in the cementless group postoperatively, although the difference was not statistically significant. The use of the femoral modular component in this series did not seem to stimulate proximal femoral bone restoration to the extent reported by other authors^11^,^26^.

This study has several limitations. Firstly, the study was a retrospective series with relatively small numbers, and the follow-ups were only short- to medium-term. However, previous studies have reported that significant differences in complication rates after cemented and uncemented implant procedures were most evident during the short- to medium-term. Secondly, we acknowledge that for measuring subsidence radiostereometric analysis is more accurate than digital radiographs, which depend on manual identification of bone landmarks. Thirdly, cases with Paprosky type IIIA, IIIB, or IV femoral bone defects were not included in this series. Therefore, we cannot conclude whether the cemented stem or the cementless modular stem is more suitable for hips with severe femoral bone deficiencies; such cases at many centers comprise a substantial number that undergo revision surgeries. Finally, although the clinical and radiologic results showed no statistical difference between the two stems, results concerning quality of life cannot be inferred from this study.

Despite these limitations, our clinical and radiologic results indicate that both the cemented fixation and cementless modular fixation can be successful for femoral revision in properly selected patients. In general, the cementless femoral fixation is the preferred choice in the femoral revision, especially in complex and complicated cases. However, the cemented femoral fixation can also be considered for older patients with minor femoral bone defects.

## Methods

### Patient data

We designed a retrospective cross-sectional study to compare the outcomes of two femoral stem designs used for revision hip arthroplasty. The Link MP prosthesis is a tapered, fluted, cementless, modular, titanium stem, while the Lubinus SP II is a wide collar, double curved, cemented, cobalt-chromium alloy stem. Between March 2004 and April 2008, 55 patients underwent revision with the cementless modular stem and 46 patients with the cemented stem at our institution. Patients with severe femoral bone defects (Paprosky type III and IV) were excluded from the study. Patients with impaction grafting were also excluded. Therefore, patients in the study were all Paprosky type I or type II^27^. Two patients with cemented stems were lost to follow-up. Finally, the study group comprised 23 patients in the cementless modular stem group and 28 patients in the cemented stem group.

In the cemented stem group, there were 13 men and 15 women, with a mean age of 68.0 years (range: 46–89 years). The original diagnoses leading to primary hip arthroplasty included osteoarthritis in 19 hips (68%), femoral neck fracture in 9 (32%). Indications for revision included aseptic loosening in 22 hips (79%) and septic loosening in 6 (21%).The average preoperative Harris Hip Score (HHS)^28^ was 46.7 (range, 33–72), and visual analog pain scale (VAPS)score was 7.1 (range, 4–9). The mean follow-up time was 6.1 years (range, 4–8 years).

In the cementless modular stem group, there were 16 men and 7 women, with a mean age of 64.3 years (range, 35–76 years). The original diagnoses leading to the primary hip arthroplasty included osteoarthritis in 15 hips (65%), femoral neck fracture in 8 (35%). Indications for revision included aseptic loosening in 15 hips (65%), and septic loosening in 8 (35%). The average preoperative HHS in this group was 41.7 (range, 24–57), and VAPS was 6.7 (range, 5–9). The mean follow-up time was 5.5 years (range, 4–8 years). The study has been approved by the Ethic Committee at Xiangya hospital, and informed consent was obtained from all patients. The use of the imaging data from the patient is permitted.

The recorded and compared preoperative characteristics of the two patient cohorts included age, gender, indications for revision, HHS, VAPS, femoral bone defect, leg length discrepancy (LLD), and follow-up duration (Table 1). The degree of femoral bone defect before revision surgery was graded by preoperative radiographs on the basis of the Paprosky classification^27^. There were no significant differences in these parameters between the two patient cohorts. (Table 1)

### Surgical technique

Two senior surgeons performed all the revision procedures for patients in the cemented stem group, through a posterolateral approach. For well-fixed stems, a 1.5- to 2-cm-wide vertical bone wedge was taken out from the area between the femoral neck and the tip of the loose femoral stem at the posterolateral femur^29^. The bone wedge was replaced and fixed by 2 cerclage cables after the femoral stem and cement was removed completely. Rasp and the trial components were used to size the femoral prosthesis appropriately. The femoral canal was thoroughly irrigated and occluded by a sturdy-fitting polythylene plug distally. High-viscosity bone cement with antibiotic (Palacos with gentamicin; Heraeus, Wehrheim, Germany) was then injected into the femoral canal with a narrow-nozzled cement gun in a retrograde direction. Careful attention was paid to creating an adequate cement mantle of 2-to 3-mm around the femoral implant. Bone grafts were not used for femoral reconstruction in this group.

For patients in the cementless modular stem group, all the procedures were performed by one senior surgeon through a posterolateral surgical approach. The wide vertical bone wedge was removed, or transfemoral osteotomy was performed, to extract the well-fixed stem and cement. Cerclage wires or cables were used to secure the bone wedge or osteotomy fragment. Once the femoral stem was removed and fibrous tissue in the femoral canal was thoroughly debrided, the femoral canal was prepared with conical reamers. An intraoperative radiograph with the last reamer *insitu* in the femoral canal was obtained to assess the canal fill and reconfirm the final stem size. The prosthesis was impacted into the reamed cavity to the planned level and rigid stability reconfirmed via axial and torsional testing. The trial proximal segment was applied and a trial reduction was performed with the stem component that was fixed distally. Once the distal fixation was achieved, a modular proximal component was chosen to optimize offset versions, leg length, neck-shaft angle, and soft tissue tension. Bone graft was not used in any of the cases of this series.

All infections in two groups were all revised in a 2-stage procedure, with infected patients receiving a minimum of 6 weeks of antibiotic treatment. The implant was not inserted until more than two weeks had elapsed after antibiotic treatment and clinical examination showed no signs of relapse of infection.

### Clinical and radiographic evaluations

All patients were clinically evaluated, and radiographs obtained preoperatively and at 6 weeks, 6 months, 1 year, and yearly thereafter. Clinical outcomes were assessed based on the Harris Hip Score (HHS)^28^, VAPS scores (range: 0–10, 0 = no pain) and incidence of thigh pain. Standard radiographs of the anteroposterior pelvis, anteroposterior and lateral of the femur were obtained for radiographic evaluation procedures. Femoral component fixation, femoral stem subsidence, LLD, femoral bone stock, and notable radiolucency were evaluated on different radiographic projections.

Cemented femoral stem fixation was assessed as described^13^, and classified as either a stable fixation, possible loosening, probable loosening, or definite loosening. Cementless stem fixation was classified as either bone ongrowth fixation, stable fibrous fixation, or unstable fixation, in accordance with the criteria of Engh et al^12^. The subsidence of the femoral component was measured using the method of Callaghan et al^30^. A vertical migration of ≥5 mm was defined as subsidence^30^,^31^. Distance from the lesser trochanter to a horizontal line drawn between two teardrops or ischial tuberosity were measured on anteroposterior views. LLD was considered corrected if the difference in length between the two sides was <5 mm^10^. Femoral bone stock was evaluated on radiographs and classified as increasing defects, constant defects, or osseous restoration, by comparing the radiographs made immediately after the hip revision and at the latest follow-up^24^. Radiolucencies were considered notable if they were ≥1 mm in ≥2 different Gruen zones^32^.

Two observers who had not participated in the primary management of the patients independently reviewed all the radiographs to minimize bias during the subjective evaluation procedure.

### Survival and statistical analyses

We performed a Kaplan-Meier survival analysis for implants till follow-up through to April 2012, taking into consideration the removal of the stem and reoperation of the hip (for any reason) as the end point. The 95% confidence interval (CI) was calculated. Continuous variables were compared using the two-sample *t*-test or paired *t*-test for independent samples; while Pearson's chi-squared (χ^2^) test was used for nominal variables. *P-*values <0.05 were considered significant. All the statistical analyses were performed using SPSS statistical software (version 15.0, SPSS, Chicago, IL). The Institution's review board approved the review of all patient records related to this study.

## Author Contributions

L.W., P.L., and Y.H. designed the study, L.W., P.L., J.X., K.L., and Z.D. collected and interpreted the data, L.W., P.L., and Y.H. wrote the main manuscript and prepared all figures. All authors reviewed the manuscript.

## Figures and Tables

**Table 1:** Preoperative characteristics of the two patient stem groups*

	Cemented (n = 28)	Cementless modular (n = 23)	*P*-value **
Age (y)	68.0 (46–89)	64.3 (35–76)	0.192
Gender, n (%)			0.097
Male	13 (46%)	16 (70%)	
Female	15 (54%)	7 (30%)	
Preoperative HHS	46.7 (33–72)	41.7 (24–57)	0.062
Preoperative VASS	7.1 (4–9)	6.7 (5–9)	0.268
Indication for revision, n (%)			0.288
Aseptic loosening	22 (79%)	15 (65%)	
Septic loosening	6 (21%)	8 (35%)	
Paprosky classification			0.753
I	7 (25%)	4 (17%)	
II	21 (75%)	19 (83%)	
LLD (mm)	3.8 (0–19)	5.7 (0.26)	0.270
LLD > 5 mm, n (%)	7 (25%)	9 (39%)	0.279
Follow-up (y)	6.1 (4–8)	5.5 (4–8)	0.112

*Values are expressed as mean (range) unless otherwise specified.

**P < 0.05 regarded as statistically significant.

**Table 2:** Follow-up outcomes of the two patient groups*

	Cemented (n = 28)	Cementless modular (n = 23)	*P*-value**
Preoperative HSS	79.2 (32–100)	83.9 (61–100)	0.195
Preoperative VAPS	0.7 (0–4)	0.5 (0–3)	0.499
Thigh pain, n (%)	1 (4%)	2(9%)	0.583
Femoral bone stock, n (%)			0.228
Bone restoration	7 (25%)	11 (48%)	
Constant defects	15 (54%)	8 (35%)	
Increasing defects	6 (21%)	4 (17%)	
Stem subsidence (mm)	2.0 (0–8.2)	1.4 (0–9.3)	0.304
Stem subsidence > 5 mm, n (%)	1 (4%)	2 (9%)	0.583
LLD (mm)	2.4 (0–14)	2.8 (0–13)	0.707
LLD > 5 mm, n (%)	5 (18%)	5 (22%)	0.739
Notable radiolucencies	4 (14%)	2 (9%)	0.678
Complications, n (%)	4	7	0.190
Intraoperative femoral fracture	0	3 (13%)	0.05
Dislocation	0	1 (4%)	0.451
Cortical perforation	2 (7%)	3 (13%)	0.647
Nerve injury	1 (4%)	0	1.000
Deep vein thrombosis	1 (4%)	0	1.000
Femoral re-revisions	1 (4%)	0	1.000
Other reoperations	2 (7%)	2 (9%)	0.588

*Values are expressed as mean (range) unless otherwise specified.

**P < 0.05 regarded as statistically significant.
